# Streptococcus agalactiae colonization in pregnant women: which culture-based detection method is best?

**DOI:** 10.1099/jmm.0.002115

**Published:** 2026-01-13

**Authors:** Sarah Schoeler, Tom Theiler, Mareike Möllers, Ioana Diana Olaru, Franziska Schuler, Frieder Schaumburg

**Affiliations:** 1 Institute of Medical Microbiology, University Hospital Münster, Münster, Germany; 2 Department of Obstetrics and Gynecology, University Hospital Münster, Münster, Germany

**Keywords:** agar, pregnancy, prenatal diagnosis, specificity and sensitivity, *Streptococcus agalactiae*

## Abstract

**Introduction.** Rectovaginal colonization with *Streptococcus agalactiae* in pregnant women is a risk factor for invasive infections of the newborns. Various culture-based approaches are available (selective vs. non-selective media±enrichment).

**Hypothesis/Gap Statement.** Comprehensive head-to-head comparisons of culture-based methods for *S. agalactiae* screening in pregnant women are largely missing.

**Aim.** We compared the test accuracy of six culture-based approaches for the detection of *S. agalactiae*.

**Methodology.** We performed a cross-sectional study on vaginal or rectovaginal swabs (in liquid Amies medium) from pregnant women. Samples were analysed in parallel in six study arms using (i) colistin nalidixic acid (CNA) agar, (ii) chromogenic *S. agalactiae* selective agar, (iii) CNA agar after enrichment in thioglycolate broth, (iv) chromogenic agar after enrichment in thioglycolate broth, (v) CNA agar after enrichment in Todd–Hewitt broth and (vi) chromogenic agar after enrichment in Todd–Hewitt broth. The test accuracy was calculated for each study arm using a composite reference standard.

**Results.** In total, 244 pregnant women were included (median age: 32 years, median weeks of pregnancy: 28 3/7). Positivity was comparable between approaches with direct inoculation on solid media (study arms i and ii, 15.6–18.0%) and with primary enrichment (study arms iii–vi, 17.6–18.9%). Each study arm had a specificity of 100%, while the sensitivity varied between 81% (CNA agar alone, study arm i) and 98% (Todd–Hewitt broth for enrichment followed by subculture on chromogenic agar, study arm vi).

**Conclusion.** Enrichment in Todd–Hewitt broth followed by subculture on chromogenic agar had the highest sensitivity (98%, specificity: 100%) for the detection of *S. agalactiae* in pregnant women.

## Data Summary

All data are made available in the manuscript. Further requests can be made to the corresponding author.

## Introduction

*Streptococcus agalactiae* belongs to group B beta-haemolytic streptococci (GBS) and is part of the rectovaginal flora. Colonization rates range between 11 and 35% with geographical variation [1]. In addition to asymptomatic colonization, *S. agalactiae* can cause a wide range of infections such as meningitis, skin and soft tissue infection and early- and late-onset sepsis not only in newborns [1] but also in women in the peri- and postpartum period [2].

Rectovaginal colonization with *S. agalactiae* in pregnant women is a risk factor for invasive *S. agalactiae* infections of the newborns (i.e. early-onset infections <7 days after delivery) with an overall incidence of 0.2–1.5 cases per 1,000 live births, irrespective of the colonization status of the mother [2,3]. Transmission of *S. agalactiae* from a colonized mother occurs in up to 4.5% of newborns [4,5]. To reduce vertical transmission, intrapartum antimicrobial prophylaxis is recommended for all mothers with *S. agalactiae* rectovaginal colonization and those with risk factors (e.g. history of a newborn with *S. agalactiae* infection, *S. agalactiae* bacteriuria) [6]. For that purpose, pregnant women should be screened for rectovaginal *S. agalactiae* colonization prior to delivery (usually 36–37 weeks of gestation) [7]. Several methods for the detection of *S. agalactiae* in rectovaginal swabs are available (e.g. PCR, culture) [8]. Culture-based approaches include selective solid media with or without selective (e.g. Todd–Hewitt broth) or non-selective enrichment broths [9,10]. Chromogenic media stain *S. agalactiae* colonies due to specific enzymatic reactions and often have a higher sensitivity compared to non-chromogenic agar [9]. The Todd–Hewitt broth was designed for the enrichment of beta-haemolysing streptococci and is often supplemented with antimicrobial agents to suppress the growth of Gram-negative bacteria [10].

It is unclear which approach and which test combination has the best performance. In particular, there is insufficient evidence to determine if a selective enrichment (e.g. Todd–Hewitt broth) is superior to a non-selective enrichment (thioglycolate broth). Therefore, the objective of this study was to compare the test performance of six different culture strategies including two selective media [colistin nalidixic acid agar (CNA) and chromogenic *S. agalactiae* selective agar] with or without *S. agalactiae* selective (Todd–Hewitt broth) or non-selective broth (thioglycolate broth).

## Methods

### Study participants

Women were recruited at the University Hospital Münster, Germany (09/2021–09/2023) and included in a cross-sectional study. Inclusion criteria were (i) pregnancy and (ii) age ≥18 years. Exclusion criteria were (i) a high probability of a medical complication triggered by the sample collection, (ii) use of antimicrobial agents within 1 week prior to sampling and (iii) duplicate samples from the same patient.

### Sample size calculation

In the absence of the known proportions of samples being positive for *S. agalactiae* in the six different culture approaches, we assumed that the test and the composite reference standard will yield positive results in the range of 15–25% [11,12]. This reflects best the expected proportion of colonization in our setting. Using the endpoints of this interval as the likely proportion of *S. agalactiae*-positive samples in the test and the composite reference standard, and setting the probability of type I error at α=0.05 and a power of 0.80, the sample size for each arm is *n*=250.

### Microbiological culture

One vaginal or rectovaginal swab (polyurethane foam-tipped in liquid Amies medium, Transwab, MWE, Corsham, England) was taken from each participant and was transported to the laboratory at room temperature within 1 h after sampling. The indication to take samples was based on the treating physicians’ judgement.

In the laboratory, swabs were vortexed, and aliquots (10 µl) of the liquid Amies medium were cultured in parallel by six different methods (Table 1). These included direct inoculation and culture on (i) CNA (Thermo Fisher Scientific, Wesel, Germany) and (ii) chromogenic *S. agalactiae* selective agar (Brilliance GBS agar, Thermo Fisher Scientific). In addition, samples were enriched in liquid medium (36 °C±1 °C for 24 h, ambient air) and subcultured on solid medium. This included (iii) CNA agar after enrichment in thioglycolate broth (Thermo Fisher Scientific), (iv) chromogenic agar after enrichment in thioglycolate broth, (v) CNA agar after enrichment in Todd–Hewitt broth (Thermo Fisher Scientific) and (vi) chromogenic agar after enrichment in Todd–Hewitt broth (Table 1). Thioglycolate broth was selected because it is one of the most widely used non-selective enrichment broths. Todd–Hewitt bouillon is a selective broth that is recommended alongside other enrichment methods (e.g. LIM bouillon) [10].

**Table 1.** Comparison of six culture-based screening approaches for the detection of *S. agalactiae* in pregnant women from vaginal and rectovaginal swabs

	Study arm					
	i	ii	iii	iv	v	vi
Primary medium	CNA	Chromogenic *S. agalactiae* selective agar	Thioglycolate broth	Thioglycolate broth	Todd–Hewitt broth	Todd–Hewitt broth
Secondary medium	Not applicable	Not applicable	CNA	Chromogenic *S. agalactiae* selective agar	CNA	Chromogenic *S. agalactiae* selective agar
Detection, % (n/total n)	15.6% (38/244)	18.0% (44/244)	17.6% (43/244)	18.4% (45/244)	18.0% (44/244)	18.9% (46/244)
Sensitivity, % (95% CI*)	81% (67–91%)	94% (82–99%)	92% (78–98%)	96% (86–100%)	94% (82–99%)	98% (89–100%)
Specificity, % (95% CI*)	100% (98–100%)	100% (98–100%)	100% (98–100%)	100% (98–100%)	100% (98–100%)	100% (98–100%)
Negative predictive value, % (95% CI*)	96% (92–98%)	99% (96–100)	98% (95–100%)	99% (96–100%)	99% (96–100%)	100% (97–100%)
Positive predictive value, % (95% CI*)	100% (91–100%)	100% (92–100%)	100% (92–100%)	100% (92–100%)	100% (92–100%)	100% (92–100%)
Hands-on time (min)†	6.12	6.12	10.33	10.33	10.33	10.33
Turnaround time of positive results (h)‡	24	24	48	48	48	48
Cost consumables [€ ($)]	€4.72 ($5.15)	€5.90 ($6.43)	€8.40 ($9.15)	€9.58 ($10.44)	€8.35 ($9.10)	€9.53 ($10.39)
Total cost [€ ($)]§	€7.05 ($7.72)	€8.23 ($9.00)	€12.33 ($13.49)	€13.50 ($14.78)	€12.28 ($13.44)	€13.46 ($14.73)

*95% confidence interval.

†Sum processing time.

‡Time between start and end of culture on solid media±prior enrichment.

§Consumables and staff.

Plates were cultured at 36 °C±1 °C for 24 h in ambient air. All suspicious colonies (i.e. grey colonies with beta-haemolysis on CNA agar or light pink colonies on *S. agalactiae* selective agar) were identified by MALDI-TOF MS (Biotyper^®^ Sirius one, Bruker, Bremen, Germany) using the compass database (MBT Compass IVD 4.2). All analyses were performed in a laboratory accredited according to DIN EN ISO 15189.

### Cost calculation

The cost calculation per arm was based on the material and personnel costs (salary x hands-on time for laboratory technicians). The hands-on time was recorded by an external person measuring the processing time with a timer.

The costs in euros (€) and US dollars ($) were calculated according to the exchange rate as of 16 October 2024. List prices per plate or broth tube (incl. value-added tax) were obtained from the manufacturer for CNA agar (€1.93; $2.10), Brilliance GBS agar (€3.11; $3.38), thioglycolate broth (€1.73; $1.88) and Todd–Hewitt broth (€1.67; $1.81). We assumed an average salary of a technician of €4,000 or $4,360 per month, respectively. Considering 21.75 working days per month and an 8 h working day, we calculate the personnel expenses to the minute (i.e. €0.38/minute or $0.42/minute).

### Statistical analysis

The sensitivity, specificity, negative predictive value and positive predictive value (incl. the 95% CI) were calculated for each study arm using a composite reference standard [13]. For that purpose, the reference is considered positive if any of the five study arms (excluding the test arm) revealed a positive result (i.e. study arm i was tested against study arms ii–vi, which were combined for the reference). The reference was considered negative if all five arms yielded a negative result. The statistical analysis was conducted using R [14].

The turnaround time of culture was defined in our study as the time from the start to the end of culture. Species identification was excluded as it might differ between laboratories using either rapid identification methods (e.g. MALDI-TOF) or culture-based approaches (e.g. Vitek2 automated systems).

## Results

A total of 244 participants were included (94 vaginal swabs, 150 rectovaginal swabs) in the final analysis. The median age was 32 years (range: 18–51), and the median number of weeks of pregnancy was 28 3/7 (range: 12 0/7–40 0/7).

Overall, *S. agalactiae* colonization was detected in 19.3% (*n*=47/244) of the women considering positive tests in any of the test arm.

The proportion of positive samples for *S. agalactiae* was comparable between approaches with direct inoculation of solid media (study arms i and ii, 15.6–18.0%) and with primary enrichment (study arms iii–vi, 17.6–18.9%, Table 1). Each study arm had a specificity of 100%, while the sensitivity was highest if Todd–Hewitt broth was used for enrichment followed by subculture on chromogenic *S. agalactiae* selective agar (study arm vi, Table 1).

The turnaround time for culture of all study arms with enrichment broth was twice as long as without an enrichment step (24 h vs. 48 h). Similarly, the total costs differed by a factor of two between the study arms (€7.05–€13.50 or $7.72–$14.78, Table 1).

## Discussion

We compared the test accuracy of six culture-based methods for the detection of *S. agalactiae* in vaginal or rectovaginal swabs from pregnant women. The main finding of our study is that a selective enrichment broth combined with a subculture on chromogenic selective solid media had the highest sensitivity for the detection of *S. agalactiae*. American guidelines recommend a selective enrichment step with a subculture on blood agar plates (corresponding to study arm v) [15]. The German guideline also recommends the use of a selective enrichment broth without a specification of the subculture medium [16]. Altogether, our results support the recommendations of these guidelines.

Although the enrichment step causes a delay of the report by 1 day, it is still acceptable in the majority of cases as *S. agalactiae* screening should be done within 5 weeks of the expected delivery day [6].

However, if rapid results are needed, a culture-based approach on a chromogenic medium without an enrichment step has an acceptable turn-around time of 24 h without a relevant decrease in sensitivity (Table 1). In case of premature rupture of membranes, PCR-based rapid tests are available for the detection of *S. agalactiae* in rectal/vaginal swabs with good sensitivity (96.6–98.5%) and specificity (99.6–100%) using culture as a reference [17,18]. In general, the sensitivity of nucleic acid amplification tests (NAAT) is higher (95.5–96.6%) compared to culture-based approaches (87.6%) [19].

One aim of *S. agalactiae* screening in pregnant women is to avoid neonatal meningitis caused by *S. agalactiae*. As with many other infectious diseases, it is not the sole presence of the pathogen that is decisive, but the inoculum [20]. For *S. agalactiae*, the infectious dose for early-onset neonatal meningitis in humans is not known, and less sensitive methods might also be appropriate to identify those women that could pose a risk for the newborn. For instance, the Centers for Disease Control and Prevention now recommend applying a reporting cut off value of ≥10^4^* S. agalactiae* c.f.u. ml^-1^ urine for intrapartum antimicrobial prophylaxis, as bacteriuria with *S. agalactiae* is an indicator for heavy vaginal colonization [6].

Considering comparable sensitivities of all study arms (except study arm i) and the rare occurrence of neonatal infection, the minimal increase in sensitivity using Todd–Hewitt broth followed by subculture on chromogenic agar (study arm vi) might not be necessarily cost-effective. From our point of view, a chromogenic *S. agalactiae* selective agar without enrichment has the best cost-to-performance ratio (Table 1). The choice of testing strategy depends on many other factors (e.g. financial resources, local epidemiology of GBS colonization and time interval from screening to delivery) and can therefore vary from setting to setting.

Our study has limitations. First, we did not apply NAAT as a gold standard. NAATs are considered more sensitive than culture-based approaches, particularly broth-enhanced (e.g. carrot broth). NAATs are considered the gold standard in some settings [21,22]. Therefore, the calculated test accuracies might be lower if a NAAT reference had been chosen. We did not include NAAT for *S. agalactiae* detection, focusing instead on culture-based methods to provide guidance for laboratories with limited resources.

Second, we did not include point-of-care tests in our study such as antigen tests or cartridge-based assays [23,24].

Third, the inclusion of additional selective broth (e.g. carrot broth and LIM broth) would have broadened the information on the best culture-based approach. Here, we focused on Todd–Hewitt broth, as it is the most frequently used selective medium in our setting.

Fourth, the study was conducted in a single tertiary referral centre which might impact the generalizability of findings to other settings and the representativeness of the population. It is likely that our population is disproportionately represented by women with pregnancy complications. Finally, the cost calculation depends on the list price and can vary between manufacturers and countries.

Future studies could consider these limitations and should have a multi-centre design including additional selective enrichment broths (e.g. LIM broth), additional selective agar media and NAAT.

In conclusion, an enrichment in Todd–Hewitt broth followed by subculture on chromogenic agar had the highest specificity (100%) and sensitivity (98%) for the detection of *S. agalactiae* in pregnant women. Several of the testing approaches had similar performance providing insights in how microbiologists could potentially simplify laboratory procedures and reduce the costs of testing.

## Abbreviations

CNA: colistin nalidixic acid
GBS: group B beta-haemolytic streptococci
NAATs: nucleic acid amplification tests
